# Integrated physiological, transcriptomic and metabolomic analysis reveals differential cold response in wheat seedlings across varieties

**DOI:** 10.3389/fpls.2026.1916983

**Published:** 2026-08-26

**Authors:** Wenjie Zheng, Peng Li, Xin Sun, Ying Liu, Mingzhu Sun, Baiqiang Yan, Zhengyong Cui

**Affiliations:** 1 Crop Research Institute, Shandong Academy of Agricultural Sciences/National Engineering Research Center of Wheat and Maize/State Key Laboratory of Wheat Improvement/Key Laboratory of Wheat Biology and Genetic Improvement in North Yellow & Huai River Valley/Shandong Provincial Technology Innovation Center for Wheat, Jinan, Shandong, China; 2 Shandong Luyan Seed Co., Ltd., Jinan, Shandong, China

**Keywords:** cold stress, integrated analysis, jasmonic acid signaling, phenylpropanoid, sibling lines

## Abstract

**Background:**

Cold stress is a major environmental constraint limiting wheat productivity worldwide. Although numerous cold-responsive pathways have been identified, the molecular basis of differential cold tolerance among genetically related wheat lines remains poorly understood. In this study, two wheat sibling lines derived from a single progeny plant of the same parental cross, Luyan951 (cold-tolerant) and Luyan955 (cold-sensitive), were employed to investigate the regulatory mechanisms of cold adaptation through integrated physiological, transcriptomic, and metabolomic analyses.

**Results:**

Physiological assays revealed that Luyan951 exhibited markedly enhanced cold tolerance, with a survival rate of 52.67% following cold treatment compared with 20.67% in Luyan955. This enhanced tolerance was accompanied by 1.90–2.41-fold greater increases in antioxidant enzyme activities (SOD, CAT, and POD) and 1.84–4.50-fold greater accumulation of proline and soluble sugars relative to Luyan955, along with substantially lower MDA accumulation. Transcriptomic and metabolomic analyses identified phenylpropanoid biosynthesis and jasmonic acid (JA) signaling as key pathways associated with cold adaptation. Compared with Luyan955, cultivar Luyan951 exhibited stronger activation of these pathways under cold stress. Key genes involved in phenylpropanoid biosynthesis (*CAD*, and *4CL*) and JA signaling (*JAZ*, *MYC2*) were significantly upregulated in Luyan951, as confirmed by qRT-PCR. Bioinformatic analyses further suggested that AP2/ERF transcription factors may act as upstream regulators of these pathways. Furthermore, subcellular localization and transcriptional activation experiments confirmed the nuclear localization and transactivation function of three AP2/ERF genes (*TraesCS5D02G318400*, *TraesCS6A02G381000*, *TraesCS6D02G366100*).

**Conclusions:**

Our findings indicate that the phenylpropanoid biosynthesis pathway plays a significant role in the cold tolerance of wheat, and together with the jasmonic acid signaling pathway, it forms a crucial regulatory network. This network promotes the scavenging of reactive oxygen species, maintains osmotic homeostasis, and stabilizes metabolism under low-temperature stress. Integrated analyses further suggest that this network may be coordinated by upstream ERF transcription factors. These findings provide comprehensive insights into the molecular mechanisms of wheat cold adaptation and offer valuable candidate genes and pathways for the genetic improvement of cold tolerance in wheat.

## 1 Introduction

As one of the most widely cultivated cereal crops worldwide, wheat (*Triticum aestivum* L.) is highly vulnerable to low-temperature stress, which can severely impair seedling establishment, growth, and grain yield. Cold injury has become an increasingly important constraint on wheat production due to the growing frequency of extreme weather events associated with climate change (Hui et al., 2025). Approximately 85% of the global winter wheat cultivation area is affected by frost damage each year, resulting in enormous economic losses (Ferrante et al., 2021; Hassan et al., 2021). Therefore, elucidating the molecular mechanisms underlying wheat cold tolerance is essential for the development of climate-resilient cultivars.

Plant responses to cold stress involve coordinated physiological and molecular processes, including osmotic regulation, reactive oxygen species (ROS) scavenging, phytohormone-mediated signaling, and ion homeostasis (Han et al., 2025; Zhuang et al., 2019). Cold stress is generally classified into two types: chilling stress (0–12 °C) and freezing stress (<0 °C) (Ding et al., 2020). The former inhibits growth and development, whereas the latter disrupts cell membranes, leading to cell death (Browse and Xin, 2001),Upon exposure to cold, plants rapidly activate complex defense mechanisms: osmoprotectants such as proline (PRO) and soluble sugars accumulate to maintain cellular integrity, while key antioxidant enzymes—superoxide dismutase (SOD), catalase (CAT), and peroxidase (POD)—are upregulated to mitigate excessive ROS and prevent oxidative damage (Bai et al., 2023; Barrera-Rojas et al., 2021). Cold receptors on the plant cell membrane perceive low-temperature signals and trigger calcium influx, thereby activating the calcium (Ca²^+^) signaling cascade network and the protein kinase-regulated phosphorylation cascade, ultimately initiating the downstream ICE1-CBF-COR signaling cascade pathway (Li et al., 2025d). Hormone-mediated regulation also plays a critical role: salicylic acid (SA) alleviates cold damage by enhancing sugar accumulation and inducing the expression of cold-responsive genes (Zhao et al., 2021), while *TaSAMT1* serves as an important regulatory node linking brassinolide (BR) and SA signaling during cold adaptation (Chu et al., 2024).

Transcription factors (TFs) function as central regulators of plant responses to environmental stress (Chunyu et al., 2025; Dawei et al., 2025; Mizoi et al., 2012; Yamaguchi-Shinozaki and Shinozaki, 2006). Plants possess a large number of TF families, whose structures and functions exhibit great diversity. They play crucial roles in growth and development as well as in stress resistance (Shaowen et al., 2026). Among these TF families, AP2/ERF, NAC, MYB, and WRKY proteins have frequently been implicated in cold adaptation through the regulation of stress-responsive gene networks (Galle et al., 2013; Li et al., 2025b, 2023; Liu et al., 2024a). Despite substantial progress in deciphering wheat cold-response pathways, how transcriptional regulation is coordinated with downstream metabolic reprogramming remains poorly understood. In particular, the contribution of phenylpropanoid metabolism and jasmonate signaling to cultivar-specific cold adaptation, as well as their upstream regulatory factors, has not been systematically investigated.

Although numerous cold-responsive genes and TFs have been identified, the regulatory consequences of these transcriptional changes at the metabolic level remain poorly understood. However, merely relying on phenotypic identification, physiological and hormonal analyses cannot fully explain the complex mechanisms of cold responses. Therefore, more in-depth studies on metabolic products and gene expression changes are needed (Wang et al., 2025b). Transcriptomics provides a comprehensive view of gene regulatory, whereas metabolomics captures downstream biochemical changes that directly reflect physiological adaptation. Integration of these two approaches has emerged as a powerful strategy for dissecting complex stress-response mechanisms (Yang et al., 2023). Conventional single-omics techniques have significant limitations in dissecting complex biological mechanisms. Metabolic-transcriptional integration analysis has been widely used to elucidate the gene-metabolite regulatory network, providing a multi-omics perspective for understanding the mechanism of plant responses to cold stress (Guo et al., 2021; Liu et al., 2024b; Wang et al., 2025a). Currently, the combined analysis of transcriptomics and metabolomics has been widely applied to various species (such as citrus, peach, alfalfa, potato and wheat), enabling cross-disciplinary exploration of the cold stress response mechanisms among multiple species and the identification of related genes and metabolites (Li et al., 2025c, 2023; Wang et al., 2022, 2023; Yu et al., 2025; Zhang et al., 2022). A major challenge in identifying cold-tolerance mechanisms is the extensive genetic variation among unrelated cultivars, which often obscures causal regulatory factors. Sibling lines with similar genetic backgrounds but contrasting stress phenotypes provide an ideal system for dissecting adaptive mechanisms because they minimize background genetic noise while maximizing trait-specific differences (Hosseini et al., 2023). Although this strategy has proven effective in several crop species, its application to wheat cold-stress research remains limited.

This study aims to utilize wheat sibling lines (Luyan951 and Luyan955) that exhibit significant differences in cold tolerance. By integrating physiological, hormonal, transcriptomic, and metabolomic analyses, the molecular basis of wheat adaptation to low temperatures will be investigated. The combination of transcriptomic, metabolomic, and physiological analysis approaches with the analysis of wheat sister lines will be employed to elucidate the potential pathways involved in the regulation of cold tolerance mechanisms. Based on the close genetic relationship between the two lines and the established roles of phenylpropanoid metabolism and jasmonic acid signaling in plant stress responses, we hypothesized that the enhanced cold tolerance of Luyan951 is driven by a more rapid and stronger activation of the phenylpropanoid biosynthesis and jasmonic acid signaling pathways, coordinated by upstream AP2/ERF transcription factors, compared with the cold-sensitive Luyan955. By uncovering these molecular and metabolic mechanisms, this study provides a theoretical foundation for the identification of cold tolerance genes and supports the development of high-yield, stress-resistant wheat varieties.

## 2 Materials and methods

### 2.1 Plant growth conditions and stress treatments

Luyan951 (951) and Luyan955 (955) are two high-yielding wheat sibling lines developed by the Crop Research Institute, Shandong Academy of Agricultural Sciences (SAAS), China. Both cultivars originated from a single F3 plant derived from the cross Luyuan502/Jimai22, and were subsequently advanced to the F15 generation through successive selfing. Genomic analysis revealed that the two lines share 96.4% SNP similarity, indicating a highly comparable genetic background. The two lines exhibit no significant differences in vegetative growth and reproductive development under normal conditions, with phenotypic divergence observed exclusively under abiotic stress. Notably, Luyan951 displays substantially greater cold tolerance than Luyan955. All plant materials were cultivated under identical conditions at the experimental station of SAAS, and seeds from the F15 generation were harvested and used for all subsequent experiments. Wheat seeds were surface-sterilized with 5% (v/v) sodium hypochlorite for 10 min, rinsed thoroughly with deionized water, and germinated on moist filter paper in darkness at 22 ± 1 °C for 48 h. Uniform seedlings were transplanted into plastic pots (10 cm x 10cm, 50 seedlings per variety) filled with a soil-vermiculite mixture (3:1, w/w). Plants were grown in a controlled growth chamber under a 14 h light/10 h dark photoperiod (light from 6:00 a.m. to 8:00 p.m.), with day/night temperatures of 22 °C/18 °C and a relative humidity of 60%. Seedlings were irrigated daily with modified Hoagland’s solution until reaching the two-leaf and one-tiller stage (Gao et al., 2024). Plants were first cold-acclimated at 4 °C for 24 h and subsequently subjected to freezing stress at −10 °C for 12 h. Leaf tissue samples were collected at 0, 3, and 6 h during freezing treatment, immediately flash-frozen in liquid nitrogen, and stored at −80 °C for subsequent physiological, transcriptomic, and metabolomic analyses. Following cold treatment, plants were recovered under control conditions (22 °C/60% RH) for 7 d to determine survival rates. All experiments were conducted using a completely randomized design with at least six independent biological replicates per treatment.

### 2.2 Quantification of physiological indicators and phytohormones

The antioxidant enzyme activities and osmotic substance concentrations were determined using commercial assay kits (Solarbio, Beijing, China). POD activity was quantified using the caffeic acid colorimetric method (BC0090), SOD activity using the nitroblue tetrazolium colorimetric method (BC5160), and CAT activity using the ammonium molybdate colorimetric method (BC0200). Further, MDA content was measured using the thiobarbituric acid method (BC0300), PRO content using the ninhydrin colorimetric method (BC0290), and soluble sugar content using the anthrone colorimetric method (BC0030).

For phytohormone analysis, 50 mg of frozen leaf tissue powder (stored at –80 °C) was homogenized in liquid nitrogen (30 Hz, 1 min). Metabolites were extracted with 1 mL methanol/water/formic acid (15:4:1, v/v/v) spiked with 10 μL internal standard mix (100 ng·mL^-^¹). After vortexing (10 min) and centrifugation (12,000 ×g, 4 °C, 5 min), the supernatants were dried under N_2_, reconstituted in 80% methanol, and filtered (0.22 μm). Analyses were performed using an ultra-performance liquid chromatography coupled with electrospray ionization tandem mass spectrometry (UPLC–ESI–MS/MS) system (ExionLC™ AD/QTRAP^®^ 6500+) equipped with a Waters ACQUITY UPLC^®^ BEH C18 column (1.7 μm, 2.1 × 100 mm). The column temperature was maintained at 40 °C, and the flow rate was 0.35 mL·min^-^¹ (Pan et al., 2010). ESI conditions were set at 550 °C, ± 5.5 kV (ion mode), and a curtain gas pressure of 35 psi. Quantitation was achieved using scheduled multiple reaction monitoring (MRM), with optimized analyte-specific declustering potential (DP)/collision energy (CE) values, calibrated against certified standards (Deng et al., 2025).

### 2.3 RNA extraction, sequencing, and transcriptome analysis

Total RNA was extracted from wheat leaf tissues using the TIANGEN RNAprep Pure Plant Plus Kit (TIANGEN, Beijing, China). RNA concentration was quantified via Qubit™ 4.0 fluorometer, and RNA integrity was assessed with an Agilent 2100 Bioanalyzer. Only samples with an RNA integrity number (RIN) ≥ 8.0 were used for subsequent analyses. Qualified RNA samples were sent to Metware Biotechnology Co., Ltd (Metware, Wuhan, China) for cDNA library preparation and 150-bp paired-end sequencing on the Illumina NovaSeq 6000 platform.

Raw sequencing reads were processed with Trim Galore (v2.2.0), which incorporates Cutadapt for adapter trimming and low-quality sequence removal (Martin, 2011). Clean reads were aligned to the wheat reference genome (*Triticum aestivum* IWGSC RefSeq genome) using HISAT2 (v2.1.0) (Kim et al., 2019). Reference-guided transcript assembly was subsequently performed using StringTie (v3.0.1) (Shinder et al., 2026) for each sample, and assembled transcripts were merged to generate a comprehensive transcript annotation containing both known and novel transcripts. The resulting transcriptome reference was then used for transcript quantification with Salmon (Srivastava et al., 2020), a transcript quantification tool demonstrated to perform well in allopolyploid species (Kuo et al., 2020), which generated transcript-level abundance estimates, including TPM values and raw counts. Differential expression analysis was performed using DESeq2 (v1.38.3) based on raw count data (Love et al., 2014). Genes with raw count ≥ 10 in at least three samples were retained to reduce noise associated with low-abundance transcripts for downstream analyses. Differentially expressed genes (DEGs) were identified using the criteria of |log_2_FC| ≥ 1 and an FDR < 0.05. Functional enrichment analysis of DEGs was performed against the Kyoto Encyclopedia of Genes and Genomes (KEGG, release 2023.01) database using the clusterProfiler R package (Xu et al., 2024). Significantly enriched pathways were identified using an adjusted P-value threshold of 0.05. Detailed sequencing statistics, quality-control metrics, and alignment rates are provided in **Supplementary Table 1**.

Weighted gene co-expression network analysis (WGCNA; v1.73) was conducted using genes with TPM > 1 in at least three samples. A soft-thresholding power of 20 was selected as the lowest value at which the scale-free topology fit index (R²) exceeded 0.80. Gene co-expression modules were subsequently identified and correlated with physiological traits and metabolite accumulation profiles (Langfelder and Horvath, 2008).

### 2.4 Gene set variation analysis and transcription factor prediction

To identify candidate upstream regulators of cold-responsive transcriptional programs, genes from the trait-associated WGCNA modules (ME2, ME5, ME7, and ME9) were submitted to WheatRegNet. Enriched transcription factor families and predicted regulatory relationships were identified using the built-in TF enrichment analysis module (Tang et al., 2023).

GSVA was performed to estimate sample-wise enrichment scores for transcription factor families and KEGG pathways associated with cold stress responses. Enrichment scores were calculated from log_2_ (TPM + 1) expression matrices using the Gaussian kernel method (kcdf = “Gaussian”, mx.diff = TRUE). Gene sets were derived from KEGG pathway annotations and wheat transcription factor family annotations obtained from PlantTFDB v5.0 (Aravind et al., 2005; Hnzelmann et al., 2013). Details of the gene sets used in this analysis were listed in **Supplementary Table 2**.

### 2.5 Metabolomic profiling

Metabolite extraction and mass spectrometry (MS) analysis were conducted by MetWare Biotechnology Co., Ltd (MetWare, Wuhan, China) following their established protocols. Briefly, 50 mg of cryodesiccated leaf tissue was extracted with 70% aqueous methanol (4 °C, 12 h) under intermittent vortex agitation (30-s pulses at 30-min intervals for 6 cycles). After centrifugation (13,200 ×g, 4 °C, 3 min), supernatants were filtered through 0.22-µm organic membranes. Chromatographic separation was achieved using an ExionLC™ AD UPLC system (SCIEX) fitted with an Agilent SB-C18 column (1.8 µm × 2.1 mm × 100 mm) under binary gradient conditions: solvent A (0.1% formic acid/water) and solvent B (0.1% formic acid/acetonitrile). Metabolite characterization utilized electrospray ionization-triple quadrupole MS/MS (SCIEX QTRAP^®^ 6500+) in dynamic MRM mode, with spectral matching against MetWare’s proprietary MWDB database v3.0.

Differentially accumulated metabolites (DAMs) were identified based on a variable importance in projection (VIP) score > 1.0 from orthogonal projections to latent structures-discriminant analysis (OPLS-DA) and an absolute fold-change threshold of |log_2_FC| ≥ 1.0. KEGG enrichment analysis was performed using those identified DAMs (Li et al., 2025a). Significantly enriched pathways were defined as those with an adjusted P-value < 0.05.

### 2.6 Correlation analysis of transcriptomic and metabolomic data

To investigate the coordinated molecular responses associated with cold tolerance, transcriptomic and metabolomic datasets were integrated based on KEGG pathway annotations. Differentially expressed genes (DEGs) and differentially accumulated metabolites (DAMs) were independently mapped to KEGG pathways, and pathways significantly enriched in both datasets were considered co-enriched. Associations between gene expression and metabolite accumulation were evaluated using Pearson correlation analysis implemented in R (v4.1.2). Pairwise Pearson correlation coefficients (PCCs) were calculated between DEGs and DAMs within significantly enriched pathways. Gene–metabolite pairs with an absolute correlation coefficient (|r|) > 0.80 and an adjusted P-value < 0.05 were considered significantly associated and were retained for network construction. Based on a predefined set of core cold-response KEGG pathways (ko00052, ko00196, ko00592, ko00940, ko00941, ko04016, and ko04075), co-enriched pathways were identified. A pathway was considered co-enriched if it showed significant enrichment in both the transcriptomic (DEGs) and metabolomic (DAMs) datasets.

### 2.7 Quantitative real-time PCR assay

Verification of RNA-seq data utilized identical RNA samples to those used for transcriptome sequencing. Quantitative PCR was performed using SYBR Green-based Super Real PreMix Plus (TIANGEN, Beijing, China) on a Roche Light Cycler^®^ 480 II system. Gene-specific primers were designed using SnapGene v6.0 and validated through NCBI Primer-BLAST (**Supplementary Table 4**). ACTIN served as the internal reference gene based on its uniform expression across treatments (|ΔCt| ≤ 0.5). Relative expression was calculated using the 2^^(−ΔΔCt)^ method with three technical replicates (Livak and Schmittgen, 2001).

### 2.8 Subcellular localization

The wheat protoplast system first isolates the mesophyll protoplasts of wheat leaves. The target gene - GFP fusion vector is transfected into the cells using PEG-mediated transformation method (Sang-Dong et al., 2007). The tobacco transient expression system first transfers the fusion vector into the *Agrobacterium* EHA105 engineering strain, injects it into the leaves of *Nicotiana tabacum*, and 48 hours later, the fluorescence signal is observed using a laser confocal microscope (excitation wavelength 488 nm). The primer sequences are listed in **Supplementary Table 4**.

### 2.9 Transcriptional activation experiments

The transcriptional activation test vector used in this study is pGBKT7, as cited in Liu’s research (Liu et al., 2022). The coding regions of *TraesCS5D02G318400*, *TraesCS6A02G381000*, and *TraesCS6D02G366100* were respectively integrated into the pGBKT7 vector using double enzyme digestion and homologous recombination methods, resulting in the construction of fusion expression vectors, pGBKT7 empty vector (negative control), and pGBKT7 -VP16 (positive control). These were respectively transformed into yeast strain Y2HGold. The transformed yeast cells were cultured at 30 °C for 3 days, and their growth status was observed. The primer sequences are listed in **Supplementary Table 4**.

### 2.10 Statistical analyses

Unless otherwise specified, all physiological and biochemical assays were performed with six independent biological replicates. For both transcriptomics and metabolomics analyses, three independent biological replicates were used. Quantitative data are presented as mean ± standard deviation (SD). Statistical significance was assessed using Student’s t-test in SPSS Statistics v26.0 (IBM^®^), with significance levels denoted as *P* < 0.05 (*) and *P* < 0.01 (**). Data organization and preliminary calculations were conducted in Microsoft^®^ Excel 16.0. Graphs and visualizations were generated using GraphPad Prism v10.0.

### 3 Results

### 3.1 Phenotypic responses to cold stress

**Figure 1:**
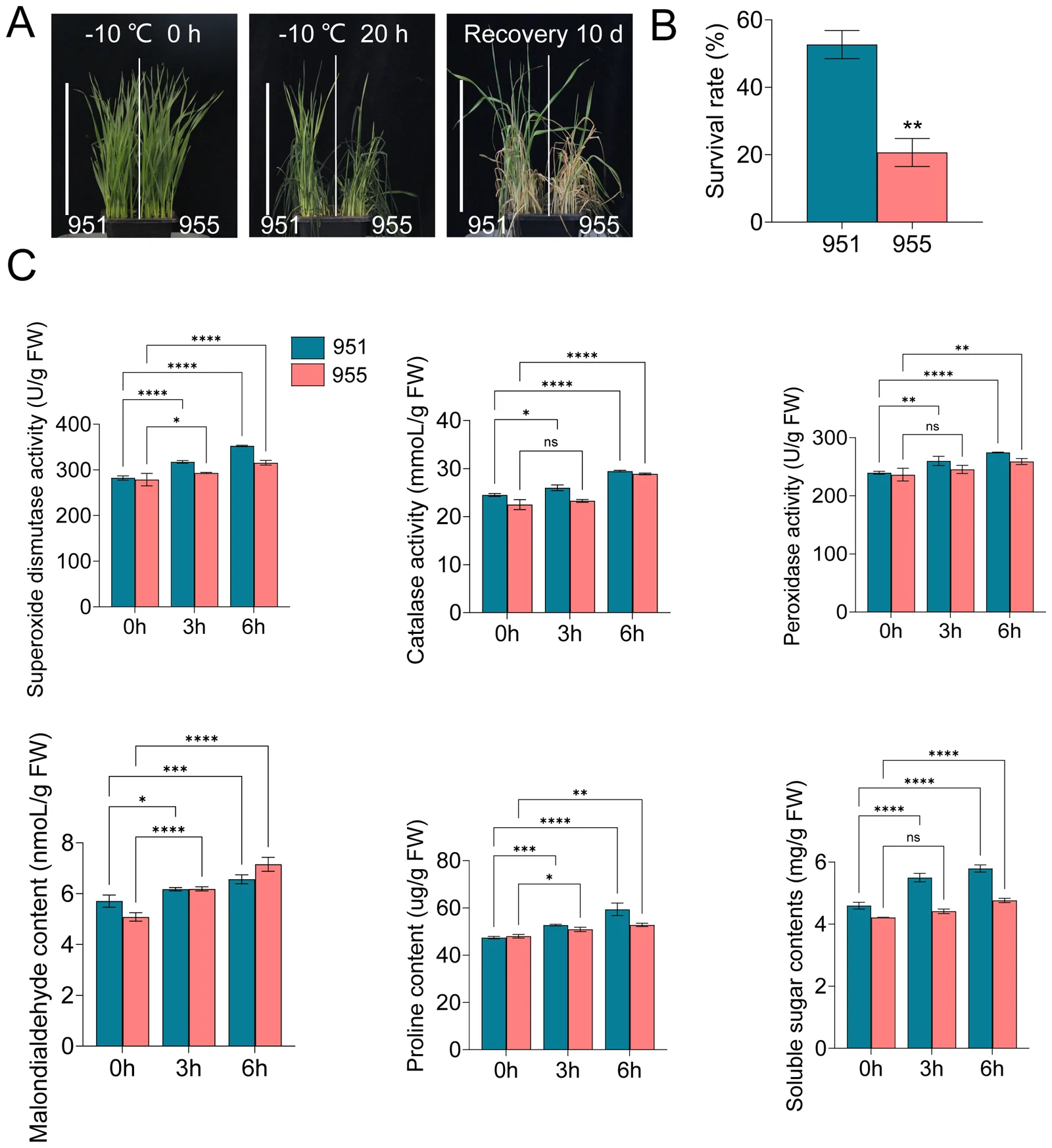
Phenotypic and physiological responses of wheat cultivars Luyan951 and Luyan955 to cold stress. **(A)** Representative phenotypes of Luyan951 and Luyan955 seedlings under cold treatment. Scale bar = 10 cm. **(B)** Survival rate (%) of Luyan951 and Luyan955 following cold stress and recovery. **(C)** Physiological responses under cold stress, including superoxide dismutase activity (U/g FW), catalase activity (mmoL/g FW), peroxidase activity (U/g FW), malondialdehyde content (nmoL/g FW), proline content (ug/g FW), and soluble sugar contents (mg/g FW). Results are presented as the mean ± standard deviation (n≥10). Statistical significance was determined by Student’s t-test **(B)** and two-way analysis of variance (ANOVA) **(C)** (*p < 0.05, **p < 0.01, ***p < 0.001 and ****p < 0.0001). 951, Luyan951; 955, Luyan955.

Seedlings of Luyan951 and Luyan955 at the two-leaf, one-tiller stage were subjected to low-temperature treatment to assess cultivar-specific cold responses. Exposure to low-temperature stress markedly inhibited plant growth and reduced post-recovery survival in both cultivars. However, Luyan951 exhibited substantially greater cold tolerance, with a survival rate of 52.67%, compared with only 20.67% in Luyan955 (**Figures 1A, B**). To further characterize the physiological differences underlying these contrasting phenotypes, key antioxidant enzyme activities and osmotic adjustment indicators were quantified. Relative to their respective untreated controls, Luyan951 displayed significant increases in SOD, CAT, and POD activities at both 3 h and 6 h post-treatment, with the magnitude of induction at 3 h being approximately 2.41-, 1.95-, and 2.26-fold greater than that in Luyan955, respectively, and this trend persisted at 6 h (1.90-, 1.54-fold for SOD and POD). In contrast, Luyan955 showed delayed activation, with statistically significant induction detected only at 6 h (**Figure 1C**). Cold stress also induced lipid peroxidation in both cultivars, as indicated by significantly elevated MDA contents at 3 h and 6 h. However, the magnitude of MDA accumulation was markedly lower in Luyan951, with the increase at 3 h and 6 h being only 0.42- and 0.41-fold of that observed in Luyan955. Similarly, PRO and soluble sugar concentrations increased significantly in both genotypes following cold exposure, reflecting enhanced osmotic adjustment. Notably, Luyan951 exhibited stronger early induction of PRO and soluble sugars, with the increase at 3 h being approximately 1.84- and 4.50-fold greater than that in Luyan955, respectively, and this advantage was maintained at 6 h (2.49- and 2.16-fold). Collectively, these results demonstrate that Luyan951 possesses a stronger antioxidant defense system and more efficient osmotic adjustment capacity under cold stress, which likely contribute to its enhanced cold tolerance and higher post-stress survival.

## 3.2 Transcriptome analysis

To elucidate the molecular mechanisms underlying differential cold tolerance, RNA-seq was performed on Luyan951 and Luyan955 under control and low-temperature conditions. Sequencing generated 304.19 Gb of nucleotide data yielding 2,045,129,286 clean reads, with an average GC content of 53.49% and high base-calling accuracy (Q20 > 96% and Q30 > 91%; **Supplementary Table 1**). A total of 18,499 DEGs were identified across all six group comparisons using the criteria described in Section 2.3 (full list in **Supplementary Table 3**).

**Figure 2:**
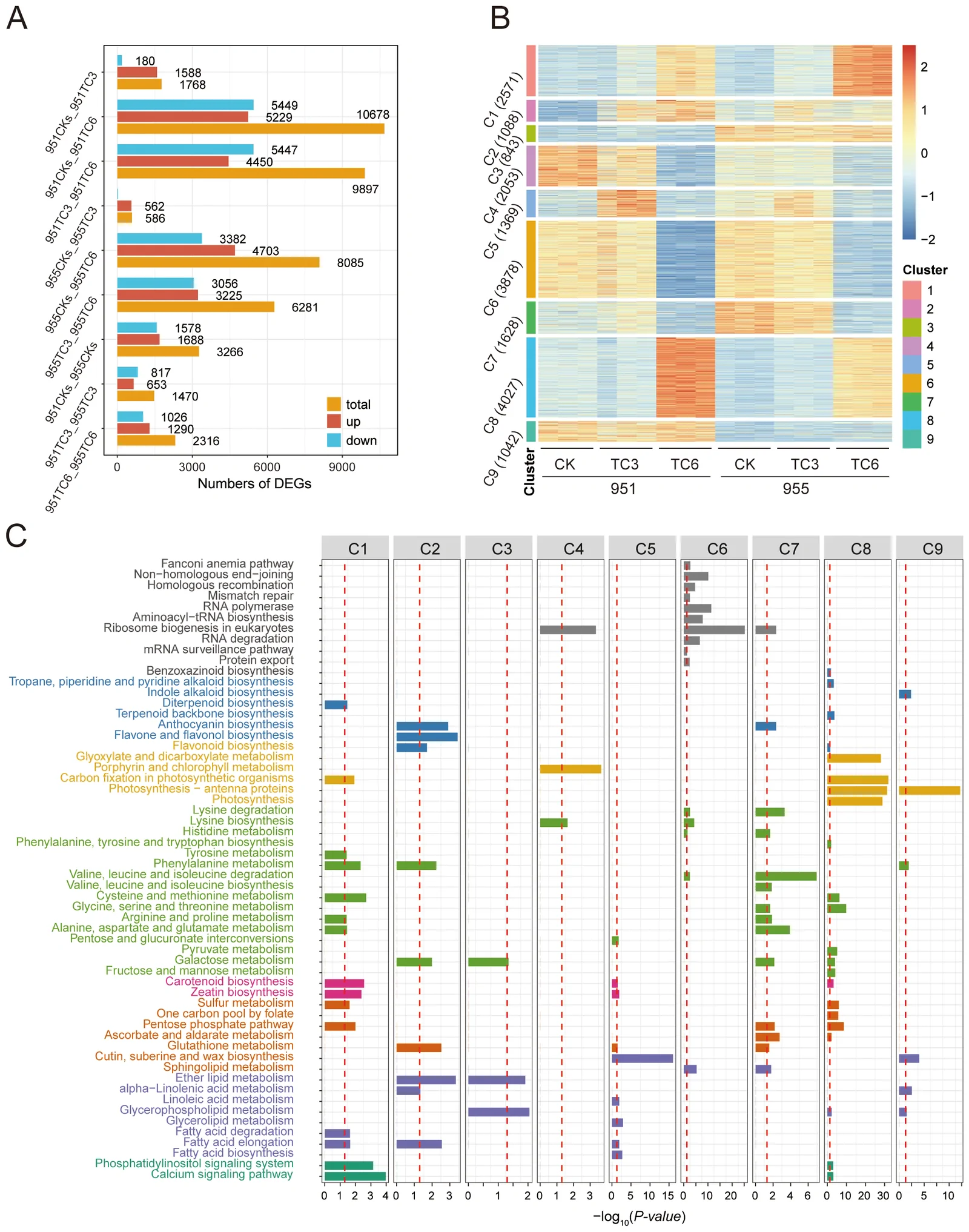
Transcriptomic responses of wheat cultivars Luyan951 and Luyan955 to cold stress. **(A)** Number of differentially expressed genes (DEGs) identified among cold treatment stages and between cultivars. **(B)** K-means clustering of DEGs into nine expression clusters (C1–C9) based on their temporal and cultivar-specific expression profiles. **(C)** KEGG enrichment analysis of genes within each cluster. Enriched pathways were categorized into cold signal perception and transduction, membrane lipid remodeling, ROS clearance and antioxidant systems, hormone biosynthesis and regulation, osmotic protection, photosynthesis and energy metabolism, and secondary metabolism and stress resistance. CK, untreated control; TC3 and TC6, cold stress treatment for 3 h and 6 h, respectively.

We compared Luyan951 and Luyan955 under both control and cold conditions. Although their genetic backgrounds are nearly identical, the gene expression patterns of the two cultivars showed substantial difference (**Figure 2A**). In both cultivars, cold treatment initially triggered predominantly the activation of transcription at TC3, as reflected by a greater number of upregulated than downregulated genes. However, the total number of DEGs at TC3 remained lower than that observed at TC6, indicating that the transcriptional response intensified with prolonged cold exposure (**Figure 2A**). Across all pairwise comparisons, Luyan951 consistently exhibited more DEGs than Luyan955, suggesting a broader transcriptional reprogramming in response to cold (**Figure 2A**).

All DEGs were clustered into nine distinct expression profiles (C1–C9; **Figure 2B**). Cluster 1 (C1) genes were predominantly upregulated at TC6, with higher expression levels observed in Luyan955 than in Luyan951. C2 genes followed a similar trend of progressive induction during cold treatment, but their expression levels were consistently higher in Luyan951 following cold exposure. C3 genes exhibited constitutively higher expression in Luyan955 and showed little change with cold. C4 genes were preferentially expressed in Luyan951 and gradually declined as cold exposure lengthened. C5 showed a transient spike at TC3, with stronger expression in Luyan951 than in Luyan955. C6 and C7 were both markedly downregulated at TC6. Although their temporal profiles were alike, C7 showed clear cultivar-specific differences. C8 showed a pattern similar to that of C1, with strong induction at TC6; however, expression levels were higher in Luyan951. C9 was constitutively higher in Luyan951 and unresponsive to cold treatment. Together, these patterns point to divergent regulatory wiring between the two cultivars: Luyan951 tends to mobilize a broader set of genes earlier, whereas Luyan955 relies more on sustained late-stage responses.

To gain the biological functions represented by these expression patterns, KEGG enrichment analysis was performed for each cluster. For ease of interpretation, significantly enriched pathways were grouped into several functional categories, including cold signal perception and transduction, membrane lipid remodeling, reactive oxygen species (ROS) scavenging and antioxidant defense, hormone biosynthesis and signaling, osmotic adjustment metabolism, photosynthesis and energy metabolism, secondary metabolism and stress resistance, as well as transcriptional regulation (**Figure 2C**). Although the nine clusters exhibited distinct temporal and genotype-dependent expression patterns, considerable functional overlap was observed among several clusters. C2 and C5, both induced at the early stage of cold treatment (TC3), were significantly enriched in membrane lipid remodeling-related pathways, suggesting rapid membrane adaptation in response to low-temperature stress. Similarly, C1, C2, and C8, which reached their highest expression levels at TC6, were commonly enriched in pathways associated with membrane lipid remodeling, ROS scavenging and antioxidant defense, osmotic adjustment metabolism, and secondary metabolism, indicating coordinated activation of multiple protective mechanisms during prolonged cold exposure. Notably, C1, which was more highly expressed in Luyan955, showed specific enrichment in calcium signaling pathways, a key component of early cold-stress perception and signal transduction. Meanwhile, C2, C5, and C8 were preferentially expressed in Luyan951 and were significantly enriched in pathways related to membrane lipid remodeling, photosynthesis and energy metabolism, and secondary metabolism. These pathways are known to contribute to cellular protection, energy maintenance, and stress adaptation under adverse conditions.

In sum, transcriptome data revealed both shared and cultivar-specific responses to cold stress. Both activated core protective pathways — membrane stabilization, antioxidant defense, osmotic regulation, and secondary metabolism, but Luyan951 displayed a stronger induction, matching its stronger cold-tolerance phenotype.

### 3.3 Co-expression network analysis reveals cultivar-specific cold-responsive modules

**Figure 3:**
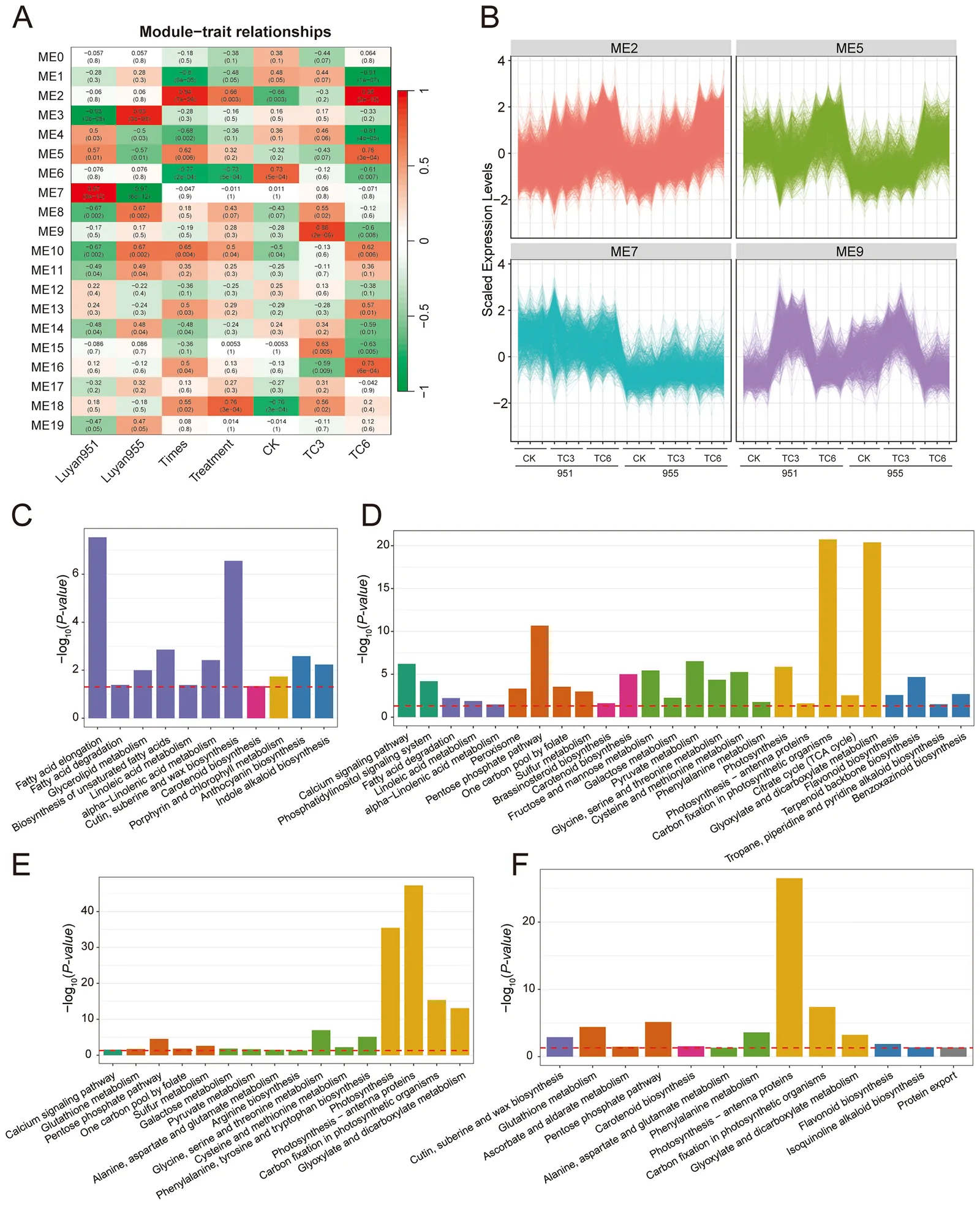
Weighted gene co-expression network analysis (WGCNA) reveals cold-responsive gene modules in wheat. **(A)** Module–trait relationship heatmap showing correlations between module eigengenes and physiological traits, treatment stages, and cultivar identity. Values indicate Pearson correlation coefficients with corresponding *P-*values. Red and green denote positive and negative correlations, respectively. **(B)** Expression profiles of representative modules associated with early cold response (ME9), late cold response (ME2), late cold response in the cold-tolerant cultivar Luyan951 (ME5), and cultivar-specific expression in Luyan951 (ME7). **(C–F)** KEGG pathway enrichment analyses of genes in ME9 **(C)**, ME2 **(D)**, ME5 **(E)**, and ME7 **(F)**.

To further investigate the relationship between gene expression patterns, cold tolerance, and cultivar-specific responses, weighted gene co-expression network analysis (WGCNA) was performed using transcriptomic data from all 18 samples. A total of 19 co-expression modules (ME1–ME19) were identified (**Figure 3A**). Several modules showed significant associations with cold treatment and/or cultivar identity (**Figure 3B**).

Among the identified modules, ME2 was strongly correlated with cold-treatment progression, particularly the TC6 stage. Genes within this module exhibited a gradual increase in expression during cold exposure in both cultivars, suggesting that ME2 represents a conserved cold-responsive transcriptional program (**Figure 3B**). The ME5 module was significantly positively associated with both the TC6 and the cold-tolerant cultivar Luyan951. Genes in this module were highly induced during prolonged cold treatment and consistently displayed higher expression levels in Luyan951 than in Luyan955. In contrast, the ME7 module was specifically associated with Luyan951 but showed little relationship with cold treatment, indicating a cultivar-dependent constitutive expression pattern. The ME9 module was significantly correlated with the early cold-response stage (TC3), with genes showing transient induction at TC3 in both cultivars and no apparent genotype-dependent differences (**Figure 3B**).

To characterize the biological functions represented by these trait-associated modules, KEGG enrichment analysis was performed on genes from ME2, ME5, ME7, and ME9. For ease of interpretation, significantly enriched pathways were grouped into several functional categories (**Figures 3C-F**). Genes in the early cold-responsive ME9 module were mainly enriched membrane lipid remodeling and membrane stability pathways, highlighting the importance of rapid membrane adaptation during the initial phase of cold stress. In contrast, the late cold-responsive ME2 module covered a broader range of biological processes, including membrane lipid remodeling, reactive oxygen species (ROS) detoxification, and secondary metabolism, indicating cold exposure triggers multiple protective mechanisms. The Luyan951-associated ME5 module were significantly enriched in pathways involved in photosynthesis and energy metabolism, osmotic adjustment, and ROS scavenging. ME7, a constitutively expressed module, showed significant enrichment of secondary metabolism and stress-resistance pathways. These results suggest that the enhanced cold tolerance of Luyan951 is associated not only with stronger cold-induced responses (ME5) but also with pre-existing transcriptional advantages (ME7), together facilitating improved energy maintenance, antioxidant defense, and stress adaptation under low-temperature conditions.

### 3.4 Metabolome analysis

**Figure 4:**
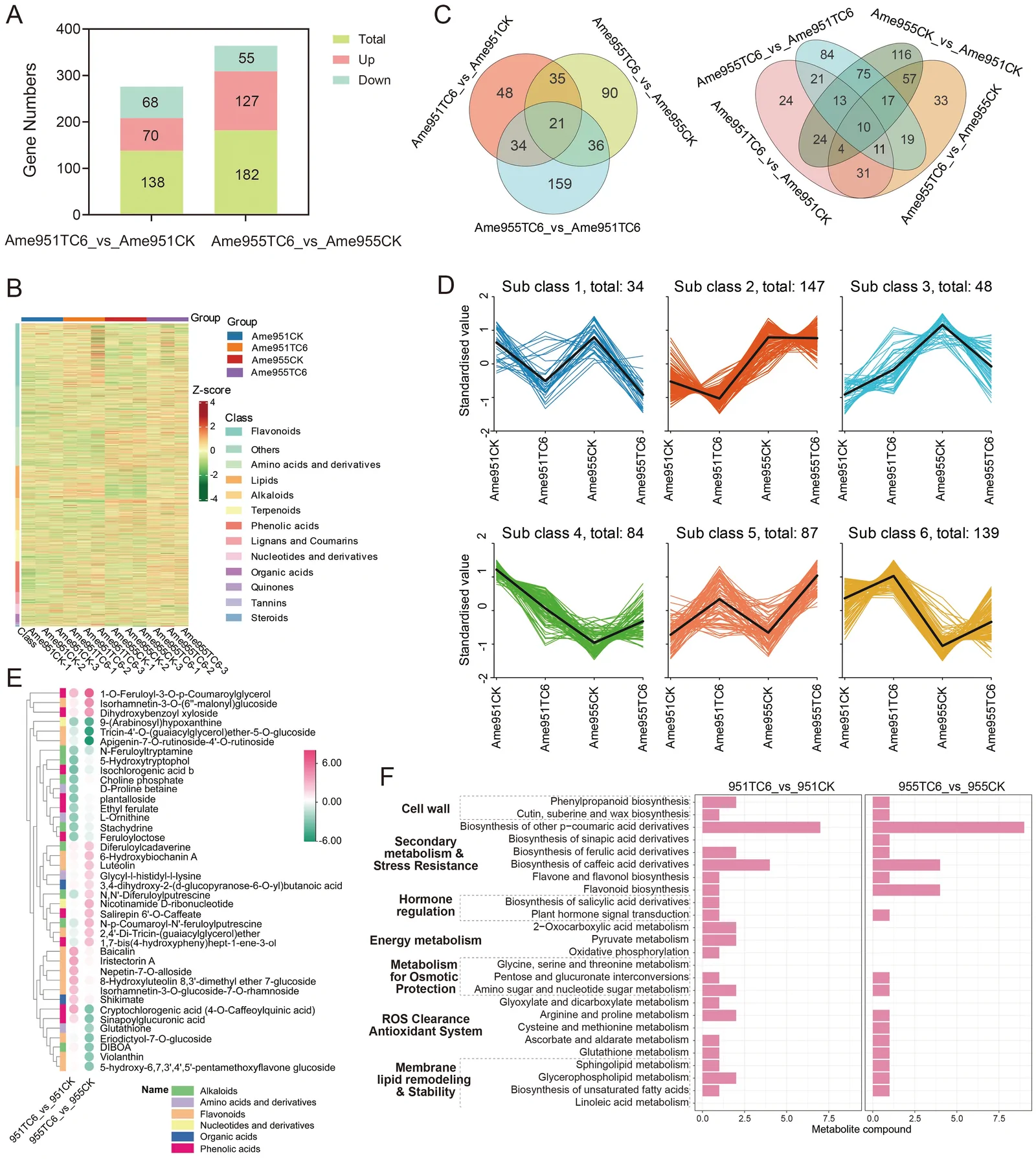
Differentially accumulated metabolites (DAMs) in Luyan951 and Luyan955 under cold stress. **(A)** Number of DAMs identified between the two wheat cultivars. **(B)** Hierarchical clustering analysis of metabolite abundance across all samples; red indicates high abundance, while green indicates low abundance. **(C)** Venn diagram showing shared and unique DAMs (false discovery rate, FDR < 0.05; fold change (FC) ≥ 2). **(D)** K-means clustering of DAMs based on accumulation profiles, partitioned into six distinct co-expression clusters. **(E)** Heat map of key metabolites responding to cold treatment in Luyan951 and Luyan955. Green indicates low-fold changes, while pink indicates high-fold changes. **(F)** KEGG pathway enrichment of differentially accumulated metabolites. The pink bars represent the number of metabolites significantly regulated within each metabolic pathway. Ame, Aestivum Metabolome; CK, untreated controls; TC6, cold stress treatment for 6 h.

To further characterize the metabolic responses underlying differential cold tolerance, widely targeted metabolomic profiling was performed on Luyan951 and Luyan955 under control and cold-stress conditions. Quality assessment of the metabolomic dataset demonstrated high reproducibility among biological replicates. Both Pearson correlation and PCA analysis clearly separated cold-treated samples from controls and distinguished the two cultivars, indicating that cold stress induced substantial metabolic reprogramming and that the metabolomic profiles were highly consistent with transcriptomic variation (**Supplementary Figure 1**-**2**). Using the criteria of VIP > 1.0 and |log_2_FC| ≥ 1.0, a total of 138 differentially accumulated metabolites (DAMs) were identified in Luyan951 following cold treatment, including 70 upregulated and 68 downregulated metabolites. In contrast, 182 DAMs were detected in Luyan955, of which 127 were upregulated and 55 were downregulated (**Figure 4A**). Hierarchical clustering analysis (HCA) based on Pearson correlation further revealed clear separation among treatment groups and cultivars, confirming that cold stress triggered distinct metabolic responses in the two wheat lines. Classification of all DAMs showed that flavonoids and amino acid derivatives constituted the predominant metabolite categories (**Figure 4B**).

### 3.5 Integrated transcriptome and metabolome analysis

**Figure 5:**
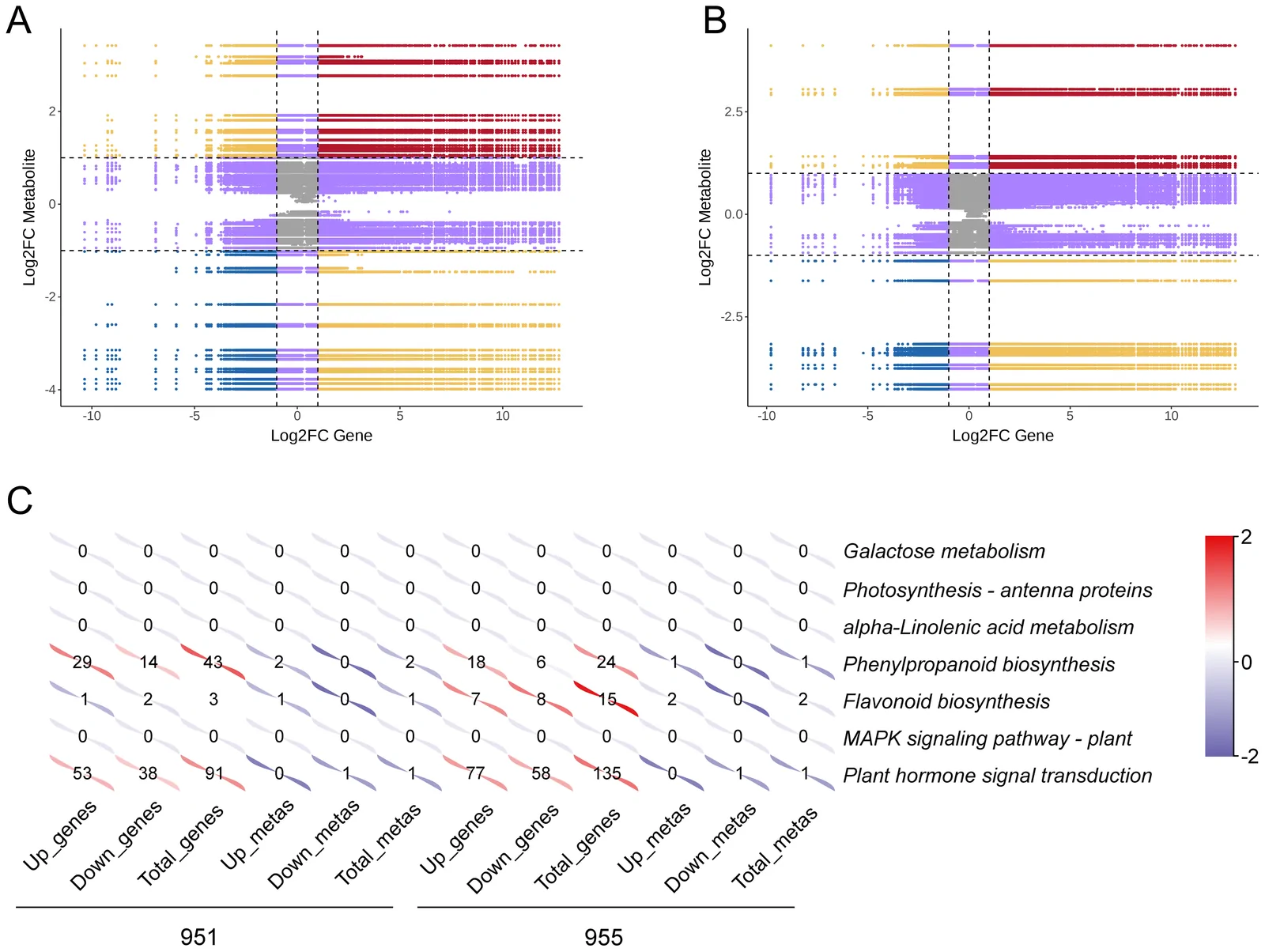
Integrated transcriptomic and metabolomic analysis of Luyan951 and Luyan955 under cold stress. **(A)** Nine-quadrant correlation analysis of DEGs and DAMs in Luyan951. **(B)** Nine-quadrant correlation analysis of DEGs and DAMs in Luyan955. Quadrants 1–9 are arranged from left to right, top to bottom. Quadrants 1, 3, 7, and 9 represent coordinated or opposite changes between gene expression and metabolite accumulation. **(C)** The heatmap displays the relative abundance patterns of DEGs and DAMs associated with key cold-responsive pathways. Blue indicates lower relative abundance, while red indicates higher relative abundance.

To elucidate the molecular mechanisms underlying cold tolerance in Luyan951 and Luyan955, integrated transcriptomic and metabolomic analyses were performed. Gene–metabolite pairs were screened within each comparison group using Pearson correlation coefficients (|r| > 0.8, *P* < 0.05). Qualified correlations were subsequently visualized using a nine-quadrant plot, which delineates fold-change patterns between corresponding genes and metabolites. Comparative analysis of quadrants 1, 3, 7, and 9, which represent coordinated or opposite changes between gene expression and metabolite accumulation, revealed significantly higher area density in Luyan951 (**Figure 5A**) than in Luyan955 (**Figure 5B**), indicating more extensive co-regulation of metabolic and transcriptional responses in the cold-tolerant cultivar. In contrast, quadrants 2, 4, 5, 6, and 8, contained gene-metabolite pairs exhibiting inconsistent fold-changes patterns. To identify pathways jointly regulated at the transcriptional and metabolic levels, integration analysis identified pathways characterized by simultaneous differential expression of DEGs and DAMs. Hierarchical clustering of these pathways (**Figure 5C**) highlighted “phenylpropanoid biosynthesis,” “plant hormone signal transduction,” and “flavonoid biosynthesis” as the most prominently enriched. Within the phenylpropanoid biosynthesis pathway, both gene expression and metabolite accumulation were substantially higher in Luyan951 than in Luyan955, indicating that the tolerant cultivar possesses a stronger capacity for metabolic regulation under low-temperature stress.

### 3.6 Phenylpropanoid biosynthesis and jasmonic acid signal transduction

**Figure 6:**
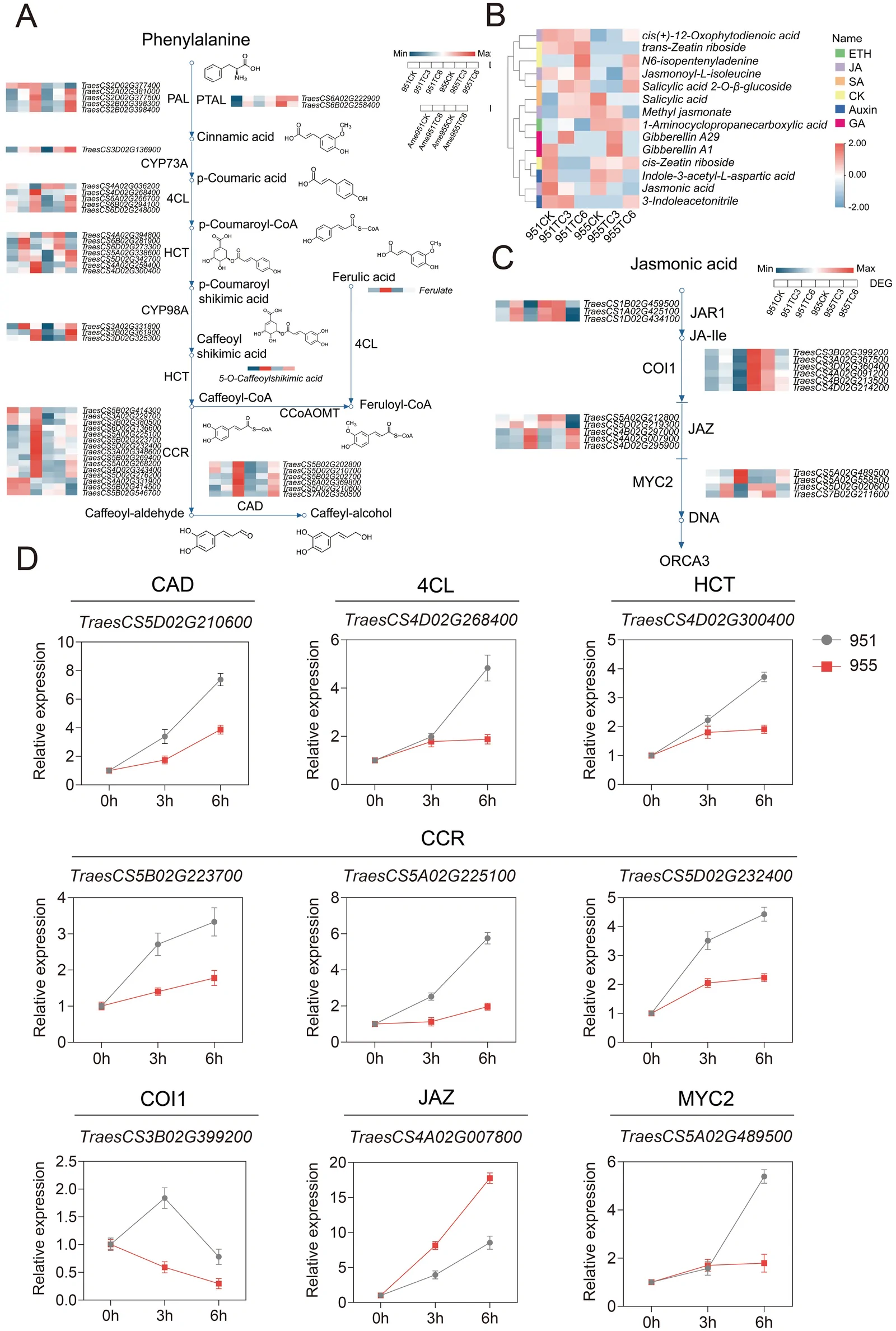
Expression profiles of the phenylpropanoid biosynthesis pathway and jasmonic acid signaling transduction pathway in wheat cultivars under cold stress. **(A)** Integrated map of phenylpropanoid biosynthesis metabolites and their corresponding key enzymes showing DEGs, DAMs and key enzymatic reaction. DEGs and DAMs were mapped onto the KEGG pathways to visualize coordinated transcriptional and metabolic changes (www.kegg.jp/kegg/kegg1.html). **(B)** Relative accumulation profiles of 14 phytohormone-related metabolites representing six major hormone classes in Luyan951 and Luyan955 following 3 h and 6 h cold treatment. **(C)** KEGG pathway map of JA signaling showing the expression patterns of DEGs associated with key signaling components. Red indicates relatively high expression levels, whereas blue indicates relatively low expression levels. **(D)** Validation of RNA-seq expression profiles by qRT-PCR analysis of nine representative cold-responsive genes involved in phenylpropanoid biosynthesis and JA signaling pathways. Expression levels were normalized to the corresponding control samples (set to 100%). Data are presented as mean ± standard deviation (SD) from at least three independent biological replicates. *TaACTIN* was used as the internal reference gene for normalization.

To further delineate the molecular basis underlying differential cold adaptation between Luyan951 and Luyan955 at the seedling stage, we examined the pathways previously identified as co-enriched through transcriptomic–metabolomic analysis. Using R-based KEGG pathway mapping, a total of 44 genes, 2 significantly altered metabolites, and 8 key enzymes were localized to the phenylpropanoid biosynthesis pathway (ko00940), with redundant branches removed for clarity. Genes encoding phenylalanine ammonia-lyase (PAL), 4-coumarate-CoA ligase (4CL), hydroxycinnamoyl transferase (HCT), cinnamoyl-CoA reductase (CCR), and cinnamyl-alcohol dehydrogenase (CAD) represented the major transcriptionally responsive components within the phenylpropanoid biosynthesis pathway (**Figure 6A**). Part of these genes exhibited stronger induction in Luyan951 than in Luyan955 under cold stress.

Quantification of phytohormone dynamics under cold stress revealed contrasting jasmonate-related responses between the two cultivars. Both cultivars exhibited similar accumulation trends for 1-aminocyclopropanecarboxylic acid (ACC), JA, jasmonoyl-L-isoleucine (JA-Ile), salicylic acid (SA), and Salicylic acid 2-O-β-glucoside (SAG). However, cis(+)-12-oxophytodienoic acid (OPDA) remained consistently elevated in Luyan951 throughout the time course, whereas Luyan955 exhibited a progressive decline. Furthermore, methyl jasmonate (MeJA) was significantly upregulated in Luyan951 but downregulated in Luyan955. All four jasmonate-related metabolites (JA, JA-Ile, OPDA, and MeJA) showed consistently higher concentrations in Luyan951 compared to Luyan955 across all time points (**Figure 6B**). To further investigate the transcriptional regulation associated with jasmonate accumulation, the JA signaling pathway (KEGG pathway ko04075) and its associated DEGs were examined. A comparative schematic (**Figure 6C**) revealed that 18 genes encoding four central functional components—jasmonic acid-amino synthetase (*JAR1*), coronatine-insensitive protein 1 (*COI1*), jasmonate ZIM domain-containing protein (*JAZ*), and TF *MYC2*—were differentially expressed between the two cultivars. These genes exhibited clear cultivar-dependent expression patterns, with generally stronger induction observed in Luyan951 than in Luyan955 under cold stress.

To validate the RNA-seq results, quantitative real-time PCR (qRT-PCR) was performed on nine representative genes involved in the phenylpropanoid biosynthesis (ko00940) and JA signaling (ko04075) pathways (**Figure 6D**). The qRT-PCR results showed strong concordance with RNA-seq–derived expression patterns for nine DEGs, with the exception of *COI1* (*TraesCS3B02G399200*), which exhibited an initial transient upregulation followed by downregulation. In contrast, *CAD* (*TraesCS5D02G210600*), *4CL* (*TraesCS4D02G268400*), *HCT* (*TraesCS4D02G300400*), *CCR* (*TraesCS5B02G223700*, *TraesCS5A02G225100*, *TraesCS5D02G232400*), *JAZ* (*TraesCS4A02G007800*), and *MYC2* (*TraesCS5A02G489500*) exhibited a progressive upregulation trend in both cultivars. Notably, the magnitude of induction was significantly higher in Luyan951. Together, these results suggest that genes such as *CAD*, *4CL*, *HCT*, *CCR*, *JAZ*, and *MYC2* may contribute to the enhanced cold tolerance of Luyan951 and thus represent promising candidates for future functional studies.

### 3.7 Identification of AP2/ERF transcription factors associated with cold tolerance

**Figure 7:**
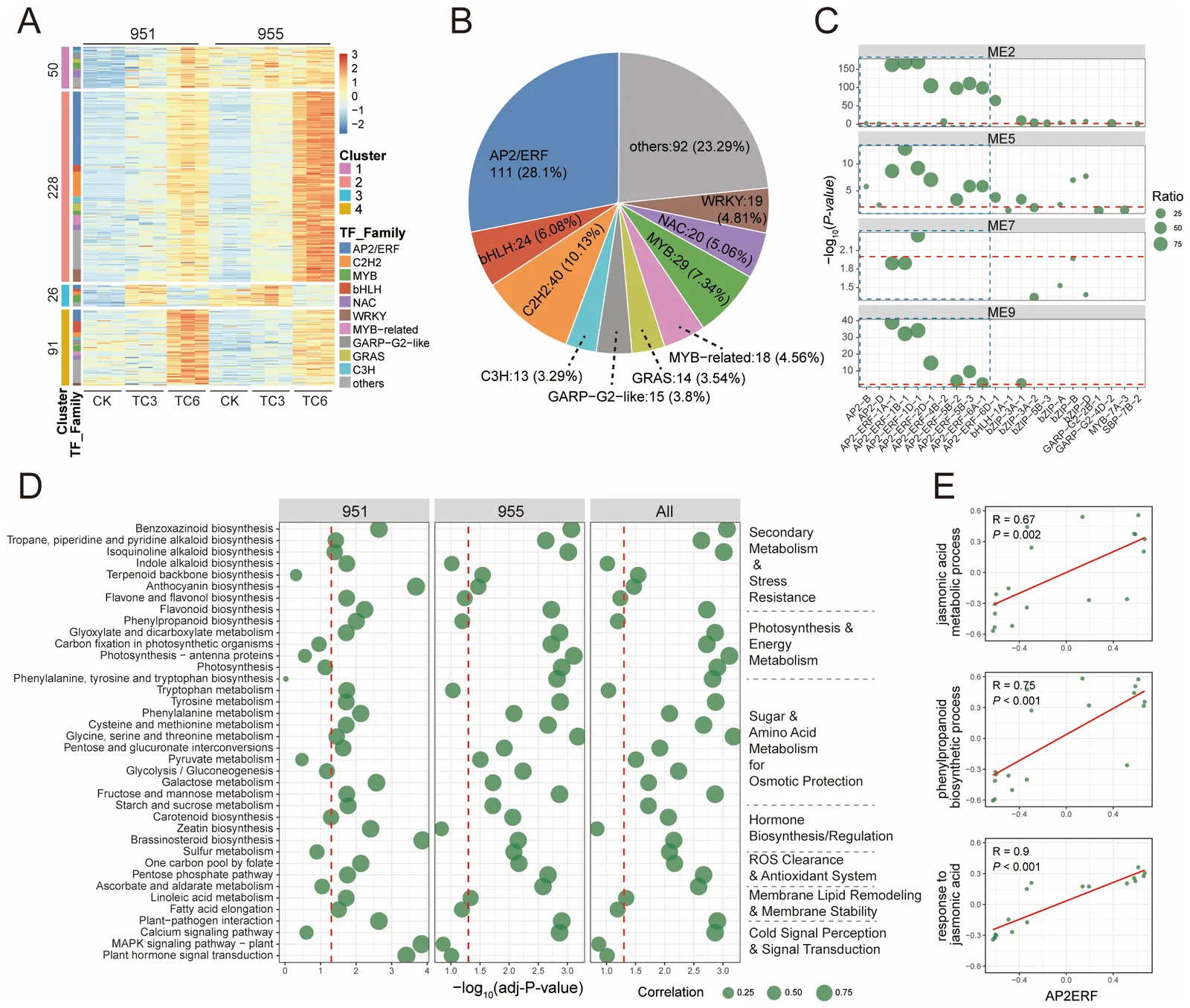
AP2/ERF transcription factors are associated with phenylpropanoid biosynthesis and jasmonate signaling during wheat cold adaptation. **(A)** Expression patterns of differentially expressed transcription factors under cold stress in Luyan951 and Luyan955. **(B)** Classification of differentially expressed transcription factor families. **(C)** Enrichment of transcription factor families predicted to regulate genes within the ME2, ME5, ME7, and ME9 co-expression modules. **(D)** Correlations between AP2/ERF family activity scores and KEGG pathway enrichment scores. **(E)** Relationships between AP2/ERF enrichment score and the phenylpropanoid biosynthesis and jasmonic acid signaling pathways.

Transcriptional regulation plays a central role in plant adaptation to abiotic stress. To identify key transcription factors (TFs) involved in wheat cold tolerance, we first analyzed TFs that were differentially expressed across cold-treatment stages and between cultivars. In total, 395 differentially expressed TF genes were identified (**Figure 7A**).

Based on their expression dynamics, these TFs were classified into four major clusters. TFs in Cluster 1 (C1) exhibited progressive induction throughout cold treatment. Clusters 2 (C2) and 4 (C4) were characterized by elevated expression during the late stage of cold stress (TC6), whereas Cluster 3 (C3) displayed transient induction during the early response phase (TC3). Notably, TFs belonging to C1, C2, and C3 generally showed higher expression levels in the cold-sensitive cultivar Luyan955, whereas C4 TFs were preferentially expressed in the cold-tolerant cultivar Luyan951 (**Figure 7A**). To further characterize the composition of cold-responsive TFs, family classification analysis was performed. Among all differentially expressed TFs, members of the AP2/ERF family represented the largest proportion (28.1%), indicating a potentially important role in regulating wheat responses to low-temperature stress (**Figure 7B**).

To identify candidate upstream regulators of cold-responsive gene networks, WheatRegNet analysis was performed using genes from the trait-associated WGCNA modules (ME2, ME5, ME7, and ME9). Remarkably, AP2/ERF family TFs were significantly enriched among the predicted regulators of all four modules (**Figure 7C**). This enrichment pattern suggests that AP2/ERF transcription factors may function as central regulators coordinating multiple cold-responsive biological processes.

To further investigate the relationship between AP2/ERF activity and cold-responsive pathways, gene set variation analysis (GSVA) was performed to estimate both AP2/ERF family activity and pathway activation scores across all samples. Correlation analysis revealed that AP2/ERF enrichment score was significantly associated with multiple metabolism-related pathways (**Figure 7D**). Notably, AP2/ERF enrichment score exhibited strong positive correlations with the phenylpropanoid biosynthesis pathway and the response to jasmonic acid pathway, both of which have established roles in plant stress adaptation (**Figure 7E**).

Taken together, these results consistently identify AP2/ERF transcription factors as key candidate regulators of wheat cold tolerance and suggest that they may mediate cold adaptation through the coordinated regulation of jasmonate signaling and phenylpropanoid metabolism.

### 3.8 Subcellular localization and transcriptional activation analysis

**Figure 8:**
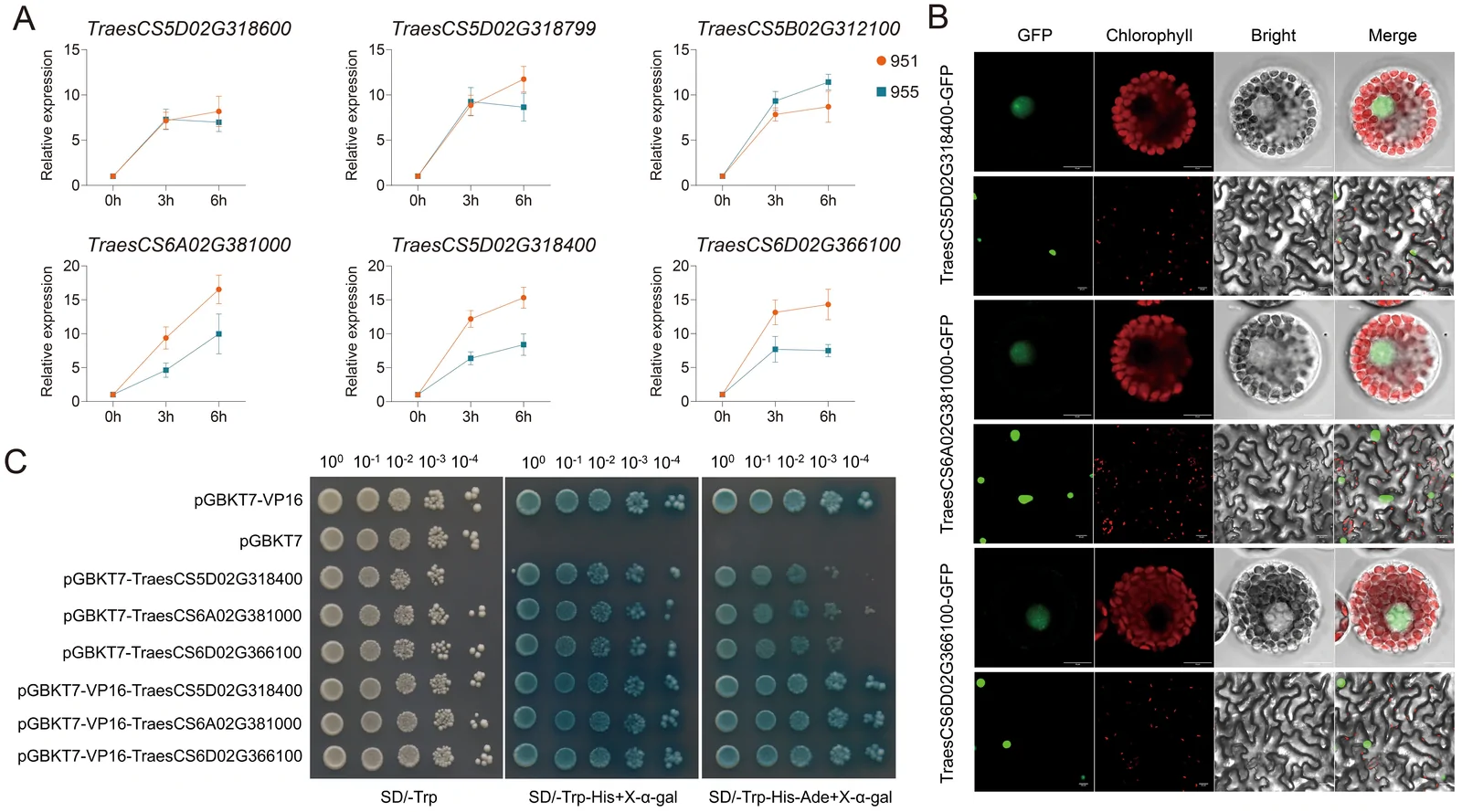
Subcellular localization and activation activity analysis. **(A)** Quantitative Real-time PCR analysis of AP2/ERF-related Genes. **(B)** Subcellular localization. **(C)** Transcriptional activation analysis.

Based on the transcriptomic analysis, WheatRegNet prediction, and GSVA results, three AP2/ERF transcription factors exhibiting strong cultivar-specific induction under cold stress were selected for further functional characterization. The quantitative validation of six significantly differentially expressed AP2/ERF genes revealed that their expression trends were consistent with the transcriptomic data. Among them, *TraesCS5D02G318400*, *TraesCS6A02G381000*, and *TraesCS6D02G366100* exhibited more pronounced differential expression under cold stress in the 951 variety (**Figure 8A**). Confocal laser scanning microscopy confirmed nuclear localization of these three genes in wheat protoplasts and tobacco epidermal cells (**Figure 8B**). Furthermore, we conducted transcriptional activation experiments. On the SD-TH+X and SD-THA+X plates, the experimental groups without the fusion positive control VP16 could grow and turn blue, while the experimental groups with the fusion VP16 could grow and their growth rate was stronger than that of pGBKT7-VP16 (**Figure 8C**). These three genes exhibited transcriptional activation activity in the yeast system. In summary, this indicates that it is involved in the transcriptional regulation of various genes in wheat under cold stress conditions.

## 4 Discussion

Among various abiotic stresses, cold stress generally suppresses the growth of winter wheat and reduces its yield during both the overwintering period and spring (Malko et al., 2023; Fang et al., 2024). Understanding the molecular mechanisms of wheat cold tolerance is crucial for improving crop resilience. While many cold-responsive pathways are known, the integrated regulatory networks remain poorly understood, especially in closely related genotypes (Liu et al., 2025; Lv et al., 2022). Using two sibling wheat lines, Luyan951 and Luyan955, with similar genetics but differing cold tolerance, we integrated multi-omics data. This revealed that coordinated changes in phenylpropanoid biosynthesis and JA signaling were strongly linked to enhanced cold tolerance. These findings provide a systems-level framework for understanding cold adaptation in wheat.

Cold stress rapidly disrupts cell redox homeostasis, resulting in excessive accumulation of ROS, and subsequent oxidative damage to membranes, proteins, and nucleic acids (Masatsugu et al., 2018; Matthew J et al., 2019). Plants therefore rely on efficient antioxidant systems and osmotic adjustment mechanisms to maintain cellular integrity under low-temperature conditions. Consistent with this, Luyan951 exhibited a higher survival rate following freezing treatment, accompanied by stronger induction of antioxidant enzyme activities (SOD, CAT, and POD) and greater accumulation of osmoprotectants, including PRO and soluble sugars. In contrast, the higher MDA content observed in Luyan955 indicates more severe membrane lipid peroxidation. Notably, antioxidant and osmotic responses were activated earlier in Luyan951 than in Luyan955, suggesting that rapid stress perception and timely deployment of defense mechanisms may be more important for cold tolerance than the absolute magnitude of stress responses (**Figure 1C**).

Transcriptomic analysis revealed distinct regulatory strategies between cultivars, with Luyan951 exhibiting more DEGs than Luyan955 under cold treatment (**Figure 2A**), indicating greater transcriptional plasticity. These DEGs were enriched in pathways for membrane lipid remodeling, ROS detoxification, osmotic regulation, and secondary metabolism (Chan et al., 2016; Guo et al., 2018). Key pathways including phenylpropanoid biosynthesis, amino sugar metabolism, unsaturated fatty acid biosynthesis, and arginine/proline metabolism were more strongly activated in Luyan951. This suggests its enhanced cold tolerance is linked to transcriptional reprogramming supporting cellular protection and metabolic adaptation.

Metabolomic profiling, which provides a direct reflection of physiological state, complements transcriptome analysis by capturing downstream biochemical changes (Hou et al., 2025). Interestingly, Luyan951 exhibited fewer differentially accumulated metabolites than Luyan955 despite showing a broader transcriptional response. Similar observations have been reported in other crops, where stress-tolerant genotypes often display more focused and efficient metabolic regulation than sensitive genotypes (Jun et al., 2019; Kira et al., 2022). One possible explanation is that extensive transcriptional regulation in Luyan951 converges on a limited number of highly effective metabolic outputs, thereby minimizing unnecessary metabolic perturbations.

Flavonoids and phenylpropanoids were key metabolites driving cultivar-specific cold responses. As antioxidants and signaling molecules, flavonoids modulate cold-responsive pathways such as CBF–COR signaling and hormone-mediated stress adaptation (Jiaxin et al., 2025; Rui et al., 2025). Consistent with this, our study revealed that Luyan951 accumulates significantly higher basal flavonoid levels than Luyan955, indicating greater stress-responsive potential. Luyan955 displayed a substantial post-stress surge in flavonoid content, whereas Luyan951 exhibited larger fold-changes (**Figure 4**), suggesting that Luyan951 relies on efficient flavonoid regulatory amplification, while Luyan955 engages in broad compensatory accumulation to reduce damage. Phenylpropanoids, synthesized via the shikimate pathway, are well-studied metabolites (Antonios et al., 2016). Previous studies have demonstrated their importance in enhancing cold tolerance in rice, Chinese cabbage, and oil-tea camellia (Shen et al., 2024; Ya-Jun et al., 2023), yet systematic evidence in wheat is still limited. In our study, both transcriptomic and metabolomic analyses revealed substantially higher enrichment of phenylpropanoid-related compounds in the cold-tolerant cultivar Luyan951 following cold exposure (**Figures 4E**, **5C**, **7D**). This provides a mechanistic basis for the strong upregulation of CAD, 4CL, HCT, and CCR observed in Luyan951 (**Figures 6A, D**), thereby underscoring the central role of the phenylpropanoid pathway in wheat cold resistance and highlighting its conserved function across plant species. To clarify the substrate preferences of the CAD/4CL/HCT/CCR homologous genes upregulated by Luyan951 and the interactions between the enzymes and metabolites, it is necessary to conduct recombinant protein expression, enzyme activity assays, and ultimately structural determination - we will conduct further research in future work.

Jasmonates are key signaling molecules that integrate environmental stress perception with downstream defense responses (Yanbing et al., 2025). Increasing evidence indicates that JA signaling positively regulates cold tolerance in diverse plant species, yet its role in wheat remains comparatively underexplored (Miao et al., 2025; Wenxin et al., 2025; Yanliang et al., 2025). In wheat, several JA-related genes—including *TaMED25*, *TaSnRK1α-TaPAP6L—*have been implicated in cold stress responses (Xia et al., 2024; Zhang, 2023), although the complete regulatory network remains poorly understood. In our study, KEGG analysis of DAMs indicated comparable enrichment in hormone signal transduction between Luyan951 and Luyan955 (**Figure 4B**), yet Luyan955 exhibited a significantly higher absolute number of DAMs (**Figure 4C**). Targeted hormone quantification confirmed elevated JA levels in Luyan951 relative to Luyan955, consistent with activation of the JA signaling pathway (**Figure 6B**). These results suggest that Luyan955 exhibits a widespread stress response with metabolic dispersion, while Luyan951 relies on precise JA-mediated regulation to effectively activate defense mechanisms. Cold stress triggers JA-Ile accumulation, facilitating COI1–JAZ interactions that target JAZ for ubiquitin–proteasome-mediated degradation, thereby releasing MYC2 to activate downstream cold-resistance responses (Ke et al., 2025). Consistently, *TraesCS3B02G399200* in Luyan951 exhibited transient upregulation followed by decline, whereas excessive accumulation of *TraesCS4A02G007800* in Luyan955 appears to impede MYC2 activation, potentially compromising cold tolerance (**Figures 6C, D**).

TFs act as central regulators that integrate environmental cues with downstream gene-expression programs. Consistent with previous studies, members of several stress-responsive TF families, including MYB, WRKY, NAC, and bZIP, were significantly represented among cold-responsive genes (Yan et al., 2025). Notably, AP2/ERF family members constituted the largest proportion of differentially expressed TFs and were enriched as predicted upstream regulators in multiple cold-associated WGCNA modules. Studies on the roles of AP2/ERF TFs in plant cold resistance remain relatively limited (Liang et al., 2024; Yihan et al., 2025), with only scattered reports in species such as rice and ginseng. Our findings demonstrate that members of the AP2/ERF family showed specific upregulation in wheat under cold stress, with representative genes such as *TraesCS5D02G318400, TraesCS6A02G381000, and TraesCS6D02G366100* displaying cultivar-specific upregulation (**Figure 8A**). Their nuclear localization and transcriptional activation activity further support their functions as bona fide transcriptional regulators. Furthermore, cold stress signals are indeed translocated from the cytoplasm to the nucleus to elicit responses and activate the expression of related genes (Kidokoro et al., 2022).

More importantly, GSVA and regulatory network analyses consistently linked AP2/ERF activity with both phenylpropanoid biosynthesis and JA signaling pathways. AP2/ERF transcription factors are well known integrators of environmental signals and hormonal responses. The enrichment of AP2/ERF regulators in multiple WGCNA modules, together with their strong association with phenylpropanoid biosynthesis and JA signaling, suggests that these TFs may function upstream of both pathways and coordinate the transcriptional reprogramming required for cold adaptation.

Taken together, our results support a model in which phenylpropanoid metabolism, jasmonate signaling, and AP2/ERF-mediated transcriptional regulation collectively contribute to cold adaptation in wheat. The use of genetically related sibling lines enabled the identification of regulatory differences that are likely associated with cold tolerance rather than broad genetic divergence. Although the present study provides comprehensive physiological and multi-omics evidence supporting the involvement of these pathways, direct genetic validation of the candidate regulators remains necessary. Future studies employing proteomics, transgenic approaches, genome editing, and large-scale germplasm validation will help establish causal relationships among AP2/ERF transcription factors, JA signaling, and phenylpropanoid metabolism. Such investigations will further advance our understanding of wheat cold adaptation and facilitate the development of climate-resilient wheat cultivars.

## 5 Conclusions

This study integrated transcriptomic, metabolomic, and physiological analysis reveals that enhanced cold tolerance in wheat cultivar Luyan951 is orchestrated by the rapid and coordinated activation of phenylpropanoid biosynthesis and JA signaling pathways. This core regulatory network, potentially governed by upstream AP2/ERF transcription factors, effectively promotes reactive oxygen species scavenging, maintains osmotic homeostasis, and stabilizes metabolism under low-temperature stress. The findings provide key molecular targets for breeding climate-resilient wheat varieties.

## Data Availability

The datasets presented in this study can be found in online repositories. The names of the repository/repositories and accession number(s) can be found in the article/**Supplementary Material**.
