# Supplementary material for: Integrated physiological, transcriptomic and metabolomic analysis reveals differential cold response in wheat seedlings across varieties
Source: Front Plant Sci. 2026 Aug 26;17:1916983. doi: 10.3389/fpls.2026.1916983 (PMC13553893; doi:10.3389/fpls.2026.1916983)
Supplement: Supplementary file 1 [file DataSheet1.zip › Supplementary Figure S1.docx]

**FIGURE S1.** Correlation analysis and principal component analysis (PCA) of transcriptome data. (A) Correlation analysis of all samples. (B) PCA plot of transcriptome results.
